# Geographical Variation in Likely Myopia and Environmental Risk Factors: A Multilevel Cross Classified Analysis of A UK Cohort

**DOI:** 10.1080/09286586.2019.1659979

**Published:** 2019-08-29

**Authors:** Tim T Morris, Jeremy A. Guggenheim, Kate Northstone, Cathy Williams

**Affiliations:** a MRC Integrative Epidemiology Unit, University of Bristol, Bristol, UK; b School of Optometry & Vision Sciences, Cardiff University, Cardiff, UK; c Bristol Medical School, University of Bristol, Bristol, UK

**Keywords:** Population density, rurality, multilevel, ALSPAC

## Abstract

***Purpose***: Previous studies have demonstrated positive associations between myopia and environmental risk factors such as urbanization. However, these have failed to account for the clustering of individuals within geographical areas, opening analyses to theoretical and statistical limitations. We demonstrate how a multilevel modelling approach can provide a more nuanced understanding of the relationship between geography and myopia. We examined longitudinal associations between onset of myopia and urban/rural status or population density.

***Methods***: Data were collected over 5 visits during an 8-year period for a UK cohort of 3,512 children. Associations between incident myopia (spherical equivalent ≤ −1.00 diopters) and both urban/rural status and population density were examined using discrete time multilevel hazard models which allow the partitioning of variance into different neighborhood and school areas.

***Results***: There was evidence for an association between myopia and higher population density (Hazard Ratio = 1.14; 95% CI = 1.032 to 1.26) after adjustment for a range of risk factors. There was no strong evidence that urban/rural status was associated with incident myopia. Only a minor amount of variation in myopia was attributable to geographical areas (<2.2%), and this was not explained by rurality or population density.

***Conclusion***: Our findings contrast with previous studies and raise the possibility that some of the results reported may have been driven by confounding bias whereby geographical differences in myopia are driven by lifestyle factors that are correlated with geographical setting.

## Introduction

The development of myopia is associated with a wide range of genetic, behavioral, social and environmental factors^1,2^ including socio-economic status^3,4^; time spent outdoors^5,6^; time spent reading^7–9^; educational attainment^10^; and geographical setting.^11–16^ While the underlying drivers for many of these factors are generally well understood, research into geographical setting remains limited. Previous studies have identified a lower prevalence of myopia amongst children living in less population-dense, rural areas compared to children in more population-dense, urban areas. Prevalence rates vary between settings and studies but these geographical patterns have been consistently observed across countries including Australia^12^, China^11,14,15^, India^17,18^, and Cambodia.^19^ Similar patterns for unaided visual acuity have also been observed whereby children in urban areas had reduced visual acuity compared to children in rural areas.^16^ The consistency of these published findings suggests a robust association between geographical setting and myopia, leading to the idea that certain aspects of geographical setting, such as town planning, could be used to improve refractive development.^12,15^

The association between geographical setting and visual outcomes could however be driven by underlying confounding factors such as socioeconomic position, education, and time spent outdoors. For example, there is uneven geographical distribution by socioeconomic status in many countries, and children are more likely to spend a greater amount of time outdoors in rural areas. Associations between geography and visual outcomes have remained robust where studies have adjusted for these factors^11,12,16^, but covariate adjustment alone fails to control for important aspects of geography. The focus on geographical setting has also been restricted to the residential setting only, with no consideration of other settings that children are exposed to. Children spend a large portion of their lives at school, and because the school and residential environments that children experience may differ, analyses that focus only on the geographical area of residence may fail to capture total geographical exposure. This issue has been overlooked in the myopia literature, despite evidence for a causal relationship between level of education and myopia.^10^

Previous studies examining geographical associations with myopia have uniformly adopted simple regression analysis, which fails to account for the natural clustering of individuals into geographical areas. This is problematic for theoretical and methodological reasons. Theoretically, using broad categories such as ‘urban’ or ‘rural’ ignores all variability *within* these categories. While there may be little variation in the most rural of areas, urban areas can vary greatly in terms of population density or the characteristics of their built environment. Methodologically, failing to account for the clustering of individuals will result in biased model parameters because people in the same areas are likely to be more similar than those from different areas. This means that standard errors will all be underestimated as study participants (observations) are not independent.^20^ Not only do they experience the *same* area, but they are likely to have been subject to similar socioeconomic and demographic sorting mechanisms which cause the non-random distribution of individuals across space. To overcome this limitation, we propose a multilevel modelling approach that (i) appropriately accounts for the clustering of individuals within geographical areas, and (ii) explicitly investigates variation across residential and school geographical environments. This approach offers a deeper understanding of the importance of geography in the development of myopia.

We use data from a UK cohort study to investigate the relationship between residential and school geographical setting, measured by urban/rural status and population density, and onset of myopia over an eight-year period of childhood. Following other studies, we hypothesize that myopia onset amongst children living in urban or densely populated areas is higher than in those living in rural or sparsely populated areas.

## Materials and methods

### Study population

Participants were children from the Avon Longitudinal Study of Parents and Children (ALSPAC). Pregnant women were eligible to enroll if they had an expected date of delivery between April 1991 and December 1992 and were resident in the (former) Avon Health Authority area in South West England. Ethical approval for the study was obtained from the ALSPAC Ethics and Law Committee and the Local Research Ethics Committees. The ALSPAC cohort is largely representative of the UK population when compared with 1991 Census data, but there was under representation of ethnic minorities, single parent families, and those living in rented accommodation. Because the ALSPAC sample has a low number of ethnic minority participants (~5%) our analysis is restricted to White children only. For full details of the cohort profile and study design see Boyd et al^21^ and Fraser et al.^22^ The study website contains details of all the data that is available through a fully searchable data dictionary and variable search tool.^23^ From the total sample of 14,775 live births, 3,512 children had data on neighborhood, school, all covariates, and at least one vision assessment.

### Myopia

All children actively participating in the ALSPAC study were asked to attend research assessments annually from the age of 7 until the age of 13, then again at 15 in which a range of physical and psychological tests were performed. Non-cycloplegic autorefraction measurements were carried out at 7, 10, 11, 12 and 15 years of age using a Canon R50 instrument (Canon USA Inc, Lake Success, NY). Subjects were classified as myopic if the mean spherical equivalent (MSE) refractive error averaged between the two eyes was less than or equal to −1.00 diopter (D). This cut point has been previously demonstrated to provide moderate sensitivity and specificity in diagnosing myopia (0.91 and 0.92 respectively).^6^ Because non-cycloplegic autorefraction was used to estimate refractive error, our measures should be interpreted as detecting “likely myopia”.

### Exposures

The 2001 Census urban/rural indicator (URI) and population density were applied to ALSPAC geographic data. The month and year of each assessment visit date was used to extract the relevant household residence from the ALSPAC address database and matched to 2001 census Lower Super Output Areas (LSOA).^24^ School addresses were obtained through linkage to the National Pupil Database and similarly matched to 2001 LSOA’s. LSOA’s contain a minimum of 40 households and 100 people and are built from clusters of neighboring postcode units; they avoid urban/rural mixes where possible and are designed to be socially homogenous while maintaining similar population counts.^25^

The URI is a settlement-based approach that separates English, Welsh and Scottish addresses by Output Area into four categories based upon population density; ‘Urban’; ‘Town and fringe’; ‘Village’; and ‘Hamlet and isolated dwelling’, before splitting each of these categories into whether surrounding areas are either ‘sparse[ly]’ or ‘less sparse[ly]’ populated to give a total of eight categories.^26^ It was designed to permit research analysis “according to different types of rural and urban area”.^26^ However, due to low counts of children in the ‘sparse’ categories, URI values were recoded to include both sparse and less sparse on each rurality category to give a total of four categories for analysis. Population density was obtained from the UK Government Office for National Statistics (ONS) and contained data on the number of people usually residing in an area and the size of the area in hectares. Population density was calculated as the number of people resident per hectare. This was transformed into units of 10 to avoid small coefficient sizes related to a one person increase in population density. Due to confidentiality issues in the data linkage, population density was unavailable for school areas.

### Covariates

A range of covariates were chosen that have been previously associated with the epidemiology of myopia. Covariates were derived from questionnaire responses completed by the child’s mother and her partner during pregnancy and through data linkage. Age was centered at each measurement occasion. Number of myopic parents (0, 1, 2) was ascertained via parents questionnaire self-reports on vision. Parents were asked to rate their vision without glasses for each eye as “always very good”; “I can’t see clearly at a distance”; “I can’t see clearly close up”; and “I can’t see much at all”. Where both eyes were categorized as “I can’t see clearly at a distance” or “I can’t see much at all” or a combination of these responses parents were classified as being myopic. Where both eyes were categorized as “always very good” or “I can’t see clearly close up” or a combination of these responses parents were classified as being non-myopic. All other combinations of responses were classified as missing. The amount of time a child spent outdoors was reported by mothers at 8 years of age in response to the question “On a weekend day, how much time on average does your child spend each day out of doors in summer” with responses categorized to either “Low” (0–3 hours) or “High” (3+ hours). The amount of time children spent reading was also reported by mothers at 8 years of age in response to the question “How much time on average does your child spend each day on average reading books for pleasure” with responses categorized to either “Low” (0–1 hours) or “High” (1+ hours). Parental Social Class based on occupation was derived from responses during pregnancy with the following responses: *I* – Professional occupations; *II* – Managerial and technical occupations; *III Non-Manual* – Skilled non-manual occupations; *III Manual* – Skilled manual occupations; *IV* – Partly-skilled occupations; and *V* – Unskilled occupations. The final two classes were combined due to low numbers. Educational attainment was measured using a General Certificate of Secondary Education (GCSE) point scores at age 16 (“Key Stage 4”; KS4), obtained through data linkage to the UK National Pupil Database. GCSE’s represent the final examinations in compulsory schooling in the UK. We use a ‘capped’ measure which is calculated as the sum of a pupil’s eight highest GCSE grades, where grades are scored in six-point integer increases from grade G = 16 to grade A* = 58, with ungraded scored as 0 (see^27^ for full information). Capped measures are preferable to uncapped measures as they prevent score inflation from simply being entered into additional subject examination. We standardized GCSE scores to have a mean of zero and standard deviation of one.

### Statistical analyses

We use discrete time multilevel models to partition longitudinal measurements into separate occasions which are clustered within individuals (see supplementary material for full details). This clustering of occasions within individuals ensures that the longitudinal measurements and their standard errors are correctly estimated. To account for the clustering of individuals into neighborhoods, another level is added into the model above the individual so that individuals are clustered into higher level areas. A normally distributed random effect is included at the area level, which allows each area to have its own regression intercept, or ‘area effect’. Allowing the impact of each area to vary is important because it permits a direct examination of empirical differences between the areas, in addition to their urban/rural and population density characteristics. Finally, we extend our models to additionally cluster individuals into their school areas. Because children from the same neighborhood do not necessarily go to the same school, and schools are not uniquely clustered within the same residential areas (that is, the two are separate but overlapping contexts), we use cross classified multilevel models.^28^ This approach allows us to cluster on neighborhood and school areas simultaneously and estimate the neighborhood and school associations separately, meaning that we can compare their impact on myopia onset and investigate the importance of each environment conditional upon the other. All results are expressed as hazard ratios with 95% confidence intervals. We fit all models using Markov Chain Monte Carlo (MCMC) Methods with 50,000 chains to ensure adequate Effective Sample Sizes^29^ and Bayesian Deviance Information Criterion’s (DIC) are provided as a measure of model fit. We present results in three sets of models. The first contains only assessment occasions, sex and age and provides the variance in myopia onset that can be attributed to neighborhood and school areas. The second includes our measures of geography; rurality and population density to assess their association with myopia onset. The third includes all covariates and provides fully adjusted associations. In all models we report the Variance Partition Coefficient (VPC) for neighbourhoods and schools, which indicate the percentage of unexplained variation at these levels. We provide fully annotated code in the supplementary material to encourage reanalysis of previous findings within a multilevel framework. Throughout we present 95% confidence intervals to discourage interpretation of results as “significant” or “non-significant”.^30,31^

## Results

### Analytical sample

Data were available for 3,512 study participants who had complete data, resulting in 12,361 person-occasion observations (mean 3.5 observations per person). Participants were distributed across 662 residential and 361 school areas. Most of the sample resided within (83.1%) and attended schools located in (82.8%) urban areas throughout the study period, consistent with the broader UK population (Table 1).^32^ Many participants were physically active (3+ hours spent outside on a weekend day in summer) and spent a low amount of time reading (0–1 hours reading per day). Engagement in the analytical sample was high, with 73.8% of participants attending at least four of the five assessments. A total of 575 of the 3512 (16.4%) participants became “likely myopic” throughout the study period, with 73 (2.4%) already classified as “likely myopic” at age 7.

**Table 1.** Sample characteristics.

	Excluded		Included	
	n	%	n	%
Sex				
Female	5,449	48.1	1,769	50.4
Male	5,891	52.0	1,743	49.6
Time outdoors				
Low	380	8.6	306	8.7
High	4,042	91.4	3,206	91.3
Time spent reading				
Low	9,416	84.9	2,236	63.7
High	1,676	15.1	1,276	36.3
Social class				
I	669	15.0	528	15.0
II	1,875	42.0	1,619	46.1
III-NM	1,105	24.7	903	25.7
III-M	574	12.9	328	9.3
IV & V	243	5.4	134	3.8
Number of myopic parents				
0	2,516	57.5	1,876	53.4
1	1,587	36.2	1,370	39.0
2	276	6.3	266	7.6
Number of assessments				
1			440	3.6
2			1,048	8.5
3			1,752	14.2
4			2,796	22.6
5			6,325	51.2
*Participant visits*				
Home rurality				
Urban	18,234	81.5	10,271	83.1
Town	1504	6.7	811	6.6
Village	1880	8.4	939	7.6
Isolated	764	3.4	340	2.8
School rurality				
Urban	40,202	84.8	10,231	82.8
Town	2700	5.7	843	6.8
Village	3409	7.2	975	7.9
Isolated	1089	2.3	312	2.5
Density	36.35	27.0	34.94	26.5

Numbers may not sum to 100% due to rounding.

### Rurality

Table 2 presents the results of the discrete time multilevel hazard models for incident myopia using categories of rurality. The VPC’s of 0.004 and 0.010 in the unadjusted model (Model 1) indicate that neighborhoods of residence and school areas accounted for only 0.4% and 1.0% of the variance in myopia respectively. Including rurality (Model 2) revealed that compared to children living in urban areas, those in areas classed as ‘Towns’ had an elevated hazard (HR: 1.28; 95% CI: 0.81, 1.97) and those in ‘Villages’ or ‘Isolated’ areas had a reduced hazard (Villages HR: 0.85; 95% CI: 0.55, 1.31; Isolated areas HR: 0.86; 95% CI: 0.45, 1.54). The school rurality estimates suggested a lower hazard for children who attended school in ‘Towns’ and ‘Villages’ (Towns HR: 0.81; 95% CI: 0.49, 1.29; Villages HR: 0.78; 95% CI: 0.5, 1.18) but an elevated hazard for those attending school in ‘Isolated’ areas (HR: 1.18; 95% CI: 0.67, 2.03). However, the point estimates for both home and school rurality were imprecise, providing no strong evidence for differences in myopia by rurality category. After including rurality the neighborhood VPC was higher than the school VPC (neighborhood VPC: 2.2%; school VPC: 0.9%). The increase in unexplained neighborhood variance from 0.4% in Model 1 implies that it is not rurality that is related to myopia onset but some other geographical factor(s) that differ between neighborhoods which we do not account for and remains unexplained by our models. It must be noted though that the error around the VPC estimates cautions against conclusive direct comparisons. Including rurality provided slightly poorer model fit as indicated by the increase in DIC from 4562 to 4565, further suggesting that rurality did not contribute to myopia onset. Controlling for covariates (Model 3) made little difference to the strength and precision of the rurality point estimates, but notably increased model fit (DIC: 4502). The variance attributable to residential neighborhoods and school areas remained minor at 2.1% and 1.3% respectively, suggesting that neighborhood geography is more important to myopia than school geography. Except for social class, all covariates were robust predictors of myopia onset. Spending time outdoors was protective of myopia while spending a high amount of time reading books and high academic achievement were risk factors. Number of myopic parents was the strongest predictor; children with two myopic parents had a hazard rate of developing myopia twice as high as those whose parents were both non-myopic.

**Table 2.** Rurality results for incident myopia.

	Model 1: unadjusted		Model 2: URI only		Model 3: Covariate adjusted	
	HR	95% CI	HR	95% CI	HR	95% CI
*Assessment occasion (ref age 7)*						
Age 10	2.17	1.63, 2.92	2.17	1.63, 2.95	2.18	1.63, 2.93
Age 11	1.53	1.1, 2.13	1.53	1.1, 2.14	1.56	1.12, 2.2
Age 12	2.27	1.68, 3.11	2.27	1.67, 3.13	2.33	1.69, 3.22
Age 15	3.53	2.63, 4.8	3.56	2.66, 4.84	3.56	2.64, 4.88
*Home rurality (ref urban)*						
Town			1.28	0.81, 1.97	1.27	0.79, 1.99
Village			0.85	0.55, 1.31	0.87	0.55, 1.32
Isolated			0.86	0.45, 1.54	0.83	0.43, 1.51
*School rurality (ref urban)*						
Town			0.81	0.49, 1.29	0.83	0.5, 1.34
Village			0.78	0.5, 1.18	0.82	0.53, 1.27
Isolated			1.18	0.67, 2.03	1.25	0.7, 2.18
Male	0.75	0.63, 0.89	0.75	0.63, 0.89	0.8	0.67, 0.95
Age (centred by occasion)	0.91	0.61, 1.34	0.91	0.61, 1.34	0.94	0.62, 1.4
Time outdoors – high (ref low)					0.64	0.49, 0.83
Reading – high (ref low)					1.4	1.18, 1.66
*Social class (ref I)*						
II					0.92	0.72, 1.17
III-NM					1.24	0.95, 1.62
III-M					1.1	0.75, 1.61
IV/V					0.91	0.51, 1.57
*Number of myopic parents (ref 0)*						
1					1.37	1.14, 1.64
2					2.06	1.55, 2.7
KS4 exam point score					1.18	1.06, 1.31
Constant	0.028	0.021, 0.035	0.028	0.021, 0.035	0.030	0.017, 0.056
*VPC’s*						
Neighbourhood VPC	0.004	0.000, 0.020	0.022	0.003, 0.052	0.021	0.001, 0.063
School VPC	0.010	0.000, 0.033	0.009	0.000, 0.029	0.013	0.001, 0.034
Bayesian DIC	4562		4565		4502	

HR, hazard Ratio. VPC, Variance Partition Coefficient; DIC, Deviance Information Criterion.

### Population density

Table 3 displays the results using population density of the residential neighborhood. There was evidence for an association between population density and onset of myopia (Table 3; Model 2), whereby children residing in more population dense areas had a higher hazard rate for becoming myopic during the study period (HR: 1.12; 95% CI: 1.03, 1.24). Each increase in 10 people resident per hectare was associated with a 14% higher Hazard Ratio of myopia, though there was evidence for non-linearity in this association as indicated by the negative density (squared) parameter. The inclusion of population density in the models led to an increase in neighborhood variation (VPC: 0.4% to 0.7%), but this was smaller than the increase when including rurality (Table 2). In contrast to the rurality models, the inclusion of population density improved model fit as indicated by the DIC (4562 to 4557). School area variation also increased by a small amount to 1.3%. Accounting for covariates did not attenuate the association between population density and myopia (Model 2 HR: 1.12; 95% CI: 1.03, 1.24; Model 3 HR: 1.14; 95% CI: 1.03,1.26), but the unexplained between-neighborhood variation as indicated by the VPC increased from 0.7% to 1.7%. This suggests that while socioeconomic and behavioral factors are important for myopia, there remain other important predictors that are geographically patterned.

**Table 3.** Population density results for incident myopia.

	Model 1: unadjusted		Model 2: Density only		Model 3: Covariate adjusted	
	HR	95% CI	HR	95% CI	HR	95% CI
*Assessment occasion* (ref age 7)						
Age 10	2.17	1.63, 2.92	2.17	1.62, 2.91	2.19	1.63, 2.92
Age 11	1.53	1.1, 2.13	1.52	1.09, 2.13	1.56	1.13, 2.18
Age 12	2.27	1.68, 3.11	2.26	1.65, 3.12	2.35	1.74, 3.2
Age 15	3.53	2.63, 4.8	3.55	2.61, 4.84	3.59	2.67, 4.81
Density			1.12	1.03, 1.24	1.14	1.03, 1.26
Density (squared)			0.99	0.98, 1	0.98	0.97, 1
Male	0.75	0.63, 0.89	0.75	0.63, 0.89	0.8	0.67, 0.96
Age (centred by occasion)	0.91	0.61, 1.34	0.9	0.6, 1.33	0.94	0.62, 1.39
Time outdoors – high (ref low)					0.63	0.48, 0.82
Reading – high (ref low)					1.39	1.16, 1.66
*Social class (ref I)*						
II					0.92	0.72, 1.19
III-NM					1.24	0.95, 1.63
III-M					1.09	0.74, 1.6
IV/V					0.89	0.49, 1.54
*Number of myopic parents (ref 0)*						
1					1.37	1.14, 1.65
2					2.05	1.53, 2.7
KS4 exam point score					1.18	1.07, 1.31
Constant	0.028	0.021, 0.035	0.024	0.017, 0.032	0.026	0.013, 0.054
*VPC’s*						
Neighbourhood VPC	0.004	0.000, 0.020	0.007	0.000, 0.027	0.017	0.001, 0.054
School VPC	0.010	0.000, 0.033	0.013	0.001, 0.039	0.008	0.000, 0.027
Bayesian DIC	4562		4557		4494	

HR, Hazard Ratio; VPC, Variance Partition Coefficient; DIC, Deviance Information Criterion.

### Sensitivity analyses

To ensure that our results were not being influenced by migration between areas, we ran a sensitivity analysis restricted only to children who did not move throughout the study period (n = 9,402 observations from 2,784 children). The results (see supplementary material) remained largely consistent with those from the full models, suggesting that migration between areas is unlikely to have biased our results.

## Discussion

Our results using a multilevel modeling approach indicated that geography was not strongly associated with onset of myopia. The variance in myopia attributable to neighborhood and school areas myopia in our UK-based sample was at most 2.2% and 1.3% respectively, suggesting that most of the differences in myopia onset were due to between individual factors. Regarding specific aspects of geographical location, we found no strong evidence for an association between myopia and rurality, but strong evidence for associations between myopia and population density. Children living in more population dense areas had a higher hazard rate for incident myopia compared to children living in less population dense areas. This association was non-linear, becoming weaker and tailing off at extreme population densities. We can only speculate on the mechanism by which population density and myopia are associated. Previous work has demonstrated that febrile illnesses are associated with myopia^33^, and their spread is likely to be higher in more population dense areas. Similarly, it is possible that the association may reflect higher pollution in more population dense areas.^34^

Adjusting for time outdoors and time spent reading^5–9^ made no change to the geographical associations, suggesting that they were not driven by differences in these factors. It is possible that population density reflects a marker of other lifestyle traits and risk factors that are not captured by our covariates. Accounting for population density did not explain underlying neighborhood variation in myopia onset, suggesting that further geographical factors also contribute to between neighborhood differences that we observed. The use of population density allowed us to investigate intra-urban effects that would not have been possible using the broader URI categories alone. Future studies focusing on population density may further highlight the importance of intra-urban effects on incident myopia. Our sensitivity analyses restricted to children who had not moved neighborhoods revealed comparable results to the main analyses, suggesting that our results were not biased by migration between different geographical settings. One interesting finding was that there was an association between time spent reading and myopia while also controlling for the association between school exam performance and myopia. This suggests that these variables do not explain the same aspects of myopiagenic risk. Given recent evidence for a causal role of education in myopia development^35,36^, this is an important consideration for future studies investigating how education predisposes children to myopia.

Our results contradict findings from other studies that have demonstrated an association between the built environment and refractive error in other countries.^11,12,14–19^ Several reasons may be responsible for this cross-context difference. It is possible that the associations observed between geographical setting and myopia in previous studies^11,12,14–19^ were confounded by lifestyle factors or socioeconomic position. If such factors are correlated with urban/rural status or population density, then associations may be upwardly biased by their exclusion as geographical factors would capture information about myopia risk factors rather than reflecting true underlying geographical effects. For example, access to and quality of education in developing countries can vary by measures of geography such as rurality^37,38^, raising the possibility that underlying correlations may have driven the associations in previous studies. Unlike previous studies, we were able to make use of longitudinal data in the form of repeat measurements. This allowed us to estimate the temporal impact of geographical areas rather than just cross-sectional associations, which are more likely to be prone to measurement error. It also meant that we were able to make full use of available data regardless of missed direct assessment visits, which would be excluded from their respective cross-sectional analyses.

The differences between our study and those in other countries may also be due to the way each country categorizes “urban” or “rural” areas, the cutoffs used for determining population density, or physical differences in landscape variation between countries. The studies in Cambodia, China, and India determined urban/rural status by broad region, while only the study in Australia used more detailed population density statistics to determine area types. Finally, it is possible that the ALSPAC cohort represents a unique group of children for whom geographical location is not important in the development of myopia. Re-analyses of previous studies within a multilevel framework that explicitly examines higher level geographical variance may elucidate the reasons underlying this difference in results. Similarly, future studies would greatly benefit from this multilevel approach.

Several limitations present in this study must be acknowledged. First, refractive error was measured by non-cycloplegic autorefraction which may have resulted in some misclassification of myopia: non-cycloplegic autorefraction is likely to overestimate myopia and is less precise than cycloplegic autorefraction in determining the timing of myopia onset.^39^ This limits the inferences that we can draw from our data and therefore further studies using the statistical approach presented here are required on samples with cycloplegic refraction data. However, there is no reason to believe that bias introduced by using non-cycloplegic autorefraction will be differential between different geographical areas. Furthermore, the use of a consistent threshold of ≤ −1.00 diopters will have limited classification bias. Despite this limitation, the trade-off between use of cycloplegia and high level of participant retention in the ALSPAC cohort offsets this limitation by providing a sample size larger than many previous studies.

Second, the study was very heavily weighted towards urban areas, with low sample sizes in the more rural categories of the URI. This restricted the power of our models and our ability to precisely estimate effect sizes. However, the use of population density as both a continuous and categorical predictor should have overcome these problems, at least in part, because it provided a more fluid measure of urbanicity than the rigid definitions imposed by administratively-set categorizations. Furthermore, sample representativeness should not bias the relationships we examine unless complex forms of associational drop out exist within the cohort.^40^

Third, our use of single point measures for certain time-varying covariates such as time spent reading or time spent outdoors relied on the assumption that such patterns did not change throughout the study period.

Fourth, we were unable to incorporate additional data on population density for the school areas due to confidentiality reasons. It is possible that this omission of data may have biased our residential population density results, though this would require inverse associations between myopia onset and residential and school population density or complex forms of confounding to be severe.

Finally, classification of parent’s myopia was based on self-reports of vision which will contain greater measurement error than clinical assessments and may lead to classification bias.

In conclusion, we found limited evidence for longitudinal associations between measures of geography and incident myopia in a UK sample. Incident myopia was associated with the population density of a child’s residential area (a 14% higher Hazard Ratio per additional 10 people resident per hectare), though this did not replicate for rurality when considering a child’s residential and school areas. Neighborhoods and schools accounted for at most 2.1% of the variation in incident myopia, and rurality and population density did not fully explain this between-context variation. This study – the first to use an appropriate multilevel modeling approach – contrasts with previous findings from a range of international settings and raises the possibility that previously observed associations may have been driven by bias due to geographical confounding.

## References

[1] Morgan I, Rose K. How genetic is school myopia? *Prog Retin Eye Res*. 2005;24(1):1–38. doi:10.1016/j.preteyeres.2004.06.004. 15555525

[2] Morgan IG, Ohno-Matsui K, Saw SM. Myopia. *Lancet*. 2012;379(9827):1739–1748. doi:10.1016/S0140-6736(12)60272-4. 22559900

[3] Saw SM, Katz J, Schein OD, Chew SJ, Chan TK. Epidemiology of myopia. *Epidemiol Rev*. 1996;18(2):175–187. http://www.ncbi.nlm.nih.gov/pubmed/9021311. 9021311 10.1093/oxfordjournals.epirev.a017924

[4] Rahi JS, Cumberland PM, Peckham CS. Myopia over the lifecourse: prevalence and early life influences in the 1958 British birth cohort. *Ophthalmology*. 2011;118(5):797–804. doi:10.1016/j.ophtha.2010.09.025. 21185080

[5] Rose KA, Morgan IG, Ip J, et al. Outdoor activity reduces the prevalence of myopia in children. *Ophthalmology*. 2008;115(8):1279–1285.doi:10.1016/j.ophtha.2007.12.019. 18294691

[6] Guggenheim JA, Northstone K, McMahon G, et al. Time outdoors and physical activity as predictors of incident myopia in childhood: a prospective cohort study. *Invest Ophthalmol Vis Sci*. 2012;53(6):2856–2865.doi:10.1167/iovs.11-9091. 22491403 PMC3367471

[7] Zylbermann R, Landau D, Berson D. The influence of study habits on myopia in Jewish teenagers. *J Pediatr Ophthalmol Strabismus*. 1993;30(5):319–322. http://www.ncbi.nlm.nih.gov/pubmed/8254449. 8254449 10.3928/0191-3913-19930901-12

[8] Saw SM, Chua WH, Hong CY, et al. Nearwork in early-onset myopia. *Invest Ophthalmol Vis Sci*. 2002;43(2):332–339. http://www.ncbi.nlm.nih.gov/pubmed/11818374. 11818374

[9] Williams C, Miller LL, Gazzard G, Saw SM. A comparison of measures of reading and intelligence as risk factors for the development of myopia in a UK cohort of children. *Br J Ophthalmol*. 2008;92(8):1117–1121. doi:10.1136/bjo.2007.128256. 18567647

[10] Mirshahi A, Ponto KA, Hoehn R, et al. Myopia and level of education: Results from the gutenberg health study. *Ophthalmology*. 2014;121(10):2047–2052.doi:10.1016/j.ophtha.2014.04.017. 24947658

[11] He M, Huang W, Zheng Y, Huang L, Ellwein LB. Refractive error and visual impairment in school children in rural southern China. *Ophthalmology*. 2007;114(2):374–382. doi:10.1016/j.ophtha.2006.08.020. 17123622

[12] Ip JM, Rose KA, Morgan IG, Burlutsky G, Mitchell P. Myopia and the urban environment: findings in a sample of 12-year-old Australian school children. *Invest Ophthalmol Vis Sci*. 2008;49(9):3858–3863. doi:10.1167/iovs.07-1451. 18469186

[13] He M, Zeng J, Liu Y, Xu J, Pokharel GP, Ellwein LB. Refractive error and visual impairment in urban children in southern china. *Invest Ophthalmol Vis Sci*. 2004;45(3):793–799. http://www.ncbi.nlm.nih.gov/pubmed/14985292. 14985292 10.1167/iovs.03-1051

[14] Saw SM, Hong RZ, Zhang MZ, et al. Near-work activity and myopia in rural and urban schoolchildren in China. *J Pediatr Ophthalmol Strabismus*. 2001;38(3):149–155. http://www.ncbi.nlm.nih.gov/pubmed/11386647. 11386647 10.3928/0191-3913-20010501-08

[15] Zhang M, Li L, Chen L, et al. Population density and refractive error among Chinese children. *Invest Ophthalmol Vis Sci*. 2010;51(10):4969–4976.doi:10.1167/iovs.10-5424. 20445117

[16] Sun H, Li A, Xu Y, Pan C. Secular trends of reduced visual acuity from 1985 to 2010 and disease burden projection for 2020 and 2030 among primary and secondary school students in china. *JAMA Ophthalmol*. 2015;133(3):262–268. doi:10.1001/jamaophthalmol.2014.4899. 25429523

[17] Padhye AS, Khandekar R, Dharmadhikari S, Dole K, Gogate P, Deshpande M. Prevalence of uncorrected refractive error and other eye problems among urban and rural school children. *Middle East Afr J Ophthalmol*. 2009;16(2):69–74. doi:10.4103/0974-9233.53864. 20142964 PMC2813593

[18] Pavithra MB, Maheshwaran R, Sujatha RM. A study on the prevalence of refractive errors among school childern of 7–15 years age group in the field practice areas of a medical college in bangalore. *Int J Med Sci Public Heal*. 2013;2(3):641–645. doi:10.5455/ijmsph.2013.220420131.

[19] Gao Z, Meng N, Muecke J, et al. Refractive error in school children in an urban and rural setting in Cambodia. *Ophthalmic Epidemiol*. 2012;19(1):16–22.doi:10.3109/09286586.2011.632703. 22273355

[20] Goldstein H. *Multilevel Statistical Models*. 4th ed. Chichester, UK: Wiley; 2011.

[21] Boyd A, Golding J, Macleod J, et al. Cohort profile: the ’children of the 90s’–the index offspring of the avon longitudinal study of parents and children. *Int J Epidemiol*. 2013;42(1):111–127.doi:10.1093/ije/dys064. 22507743 PMC3600618

[22] Fraser A, Macdonald-wallis C, Tilling K. et al. Cohort profile: the avon longitudinal study of parents and children: ALSPAC mothers cohort. *Int J Epidemiol*. 2013;42:97–110. doi:10.1093/ije/dys066. 22507742 PMC3600619

[23] ALSPAC. ALSPAC data dictionary. Web site. http://www.bristol.ac.uk/alspac/researchers/our-data/. Published 2014.

[24] Office for National Statistics. Super output areas (SOAs). Web site. http://www.ons.gov.uk/ons/guide-method/geography/beginner-s-guide/census/super-output-areas–soas-/index.html. Published 2014.

[25] Office for National Statistics. Output Areas (OA). Web site. http://www.ons.gov.uk/ons/guide-method/geography/beginner-s-guide/census/output-area–oas-/index.html. Published 2013.

[26] Office for National Statistics. ONS postcode directory user guide. Web site. https://geoportal.statistics.gov.uk/geoportal/catalog/main/home.page. Published 2012.

[27] Department for Education. *National Pupil Database Key Stage 5 User Guide,*London, UK. 2011.

[28] Goldstein H. Multilevel cross-classified models. *Sociol Methods Res*. 1994. doi:10.1177/0049124194022003005.

[29] Browne W *MCMC Estimation in MLwiN* Version *2.32*.; 2015.

[30] Colquhoun D, London C. An investigation of the false discovery rate and the misinterpretation of P values. *R Soc Open Sci*. 2014;1(3):1–15. doi:10.1098/rsos.140216. PMC4448847 26064558

[31] Sterne JAC, Cox DR, Smith GD. Sifting the evidence - what is wrong with significance tests? Another comment on the role of statistical methods. *BMJ*. 2001;322(7280):226–231. doi:10.1136/bmj.322.7280.226. 11159626 PMC1119478

[32] Office for National Statistics. *Rural and Urban Area Classification 2004. An Introductory Guide*. London; 2005. Retrieved from https://www.gov.uk/government/uploads/system/uploads/attachment_data/file/239082/2001-rural-urban-definition-methodology-intro.pdf.

[33] Guggenheim JA, Williams C. Childhood febrile illness and the risk of myopia in UK Biobank participants. *Eye*. 2016. doi:10.1038/eye.2016.7. PMC4834038 26846593

[34] Dadvand P, Nieuwenhuijsen MJ, Basagaña X, et al. Traffic-related air pollution and spectacles use in schoolchildren. *PLoS One*. 2017. doi:10.1371/journal.pone.0167046. PMC5378327 28369072

[35] Cuellar-Partida G, Lu Y, Kho PF, et al. Assessing the genetic predisposition of education on myopia: a mendelian randomization study. *Genet Epidemiol*. 2016;40(1):66–72.doi:10.1002/gepi.21936. 26497973 PMC4915592

[36] Mountjoy E, Davies NM, Plotnikov D, et al. Education and myopia: assessing the direction of causality by mendelian randomisation. *BMJ*. 2018. doi:10.1136/bmj.k2022. PMC5987847 29875094

[37] Connolly S, O’Reilly D, Rosato M. Increasing inequalities in health: Is it an artefact caused by the selective movement of people? *Soc Sci Med*. 2007;64(10):2008–2015. doi:10.1016/j.socscimed.2007.02.021. 17379374

[38] Kingdon GG. The progress of school education in India. *Oxford Rev Econ Policy*. 2007;23(2):168–195. doi:10.1093/oxrep/grm015.

[39] Williams C, Miller L, Northstone K, Sparrow JM. The use of non-cycloplegic autorefraction data in general studies of children’s development. *Br J Ophthalmol*. 2008. doi:10.1136/bjo.2007.136051. 18441189

[40] Rothman KJ, Gallacher JEJ, Hatch EE. Why representativeness should be avoided. *Int J Epidemiol*. 2013;42(4):1012–1014. doi:10.1093/ije/dys223. 24062287 PMC3888189

